# Dexmedetomidine-flurbiprofen axetil-based opioid-free analgesia attenuates postoperative melatonin suppression and improves sleep quality after thyroidectomy: a randomized controlled trial

**DOI:** 10.3389/fphar.2026.1839440

**Published:** 2026-07-02

**Authors:** Rui Guo, Xin Luo, Li Chen, Pan-Guo Rao, Li-Feng Wang, Xing-Heng Lei, Guang-Yu Dai, Hao Fang

**Affiliations:** 1 Department of Anesthesiology, First Affiliated Hospital of Gannan Medical University, Ganzhou, Jiangxi, China; 2 Ganzhou Key Laboratory of Anesthesiology, Ganzhou, China; 3 The First Clinical Medical College of Gannan Medical University, Ganzhou, China; 4 Department of Anesthesiology, Heyuan People’s Hospital, Heyuan, Guangdong, China

**Keywords:** circadian rhythm, dexmedetomidine, melatonin, opioid-free analgesia, sleep quality, thyroidectomy

## Abstract

**Objective:**

To determine whether an opioid-free patient-controlled intravenous analgesia (PCIA) regimen based on dexmedetomidine and flurbiprofen axetil improves postoperative sleep quality and affects nocturnal melatonin secretion compared to a sufentanil-based regimen in patients undergoing thyroidectomy.

**Methods:**

In this prospective, randomized, double-blind controlled trial, 96 patients undergoing thyroidectomy were randomly assigned to receive either opioid-free PCIA (dexmedetomidine, flurbiprofen axetil, and ondansetron) or opioid PCIA (sufentanil and ondansetron). PCIA was initiated 5 min before the end of surgery. The primary outcome was postoperative sleep quality, assessed using the Richards-Campbell Sleep Questionnaire (RCSQ). Secondary outcomes included urinary 6-sulfatoxymelatonin (6-SMT) excretion normalized to creatinine, detailed sleep parameters, anxiety levels, pain intensity, sedation levels, and postoperative adverse events. Assessments of sleep quality, urinary 6-SMT excretion, and anxiety levels were performed preoperatively (T0) and on postoperative days 1 (T1) and 2 (T2). Pain intensity (assessed by the Visual Analog Scale, VAS) and sedation levels (assessed using the Ramsay Sedation Scale) were measured at postoperative hours 1, 6, 24, and 48.

**Results:**

Patients in the opioid-free group exhibited significantly higher RCSQ scores at T1 and T2, indicating improved postoperative sleep quality (all *P* < 0.001). Correspondingly, urinary 6-SMT excretion was significantly higher in the opioid-free group at both postoperative time points (*P* < 0.001), suggesting better preservation of nocturnal melatonin secretion. Detailed sleep parameters showed shorter sleep latency, fewer nocturnal awakenings, and longer total sleep time in the opioid-free group (all *P* < 0.01). Anxiety levels were significantly lower in the opioid-free group (P < 0.001). Postoperative pain intensity and sedation levels were comparable between groups at all time points (all *P* > 0.05). The incidences of nausea, vomiting, and pruritus were significantly reduced in the opioid-free group (*P* < 0.05).

**Conclusion:**

An opioid-free PCIA regimen based on dexmedetomidine and flurbiprofen axetil provides non-inferior postoperative analgesia while attenuating postoperative melatonin suppression, improving sleep quality, reducing anxiety, and decreasing opioid-related adverse events. This opioid-sparing strategy may represent an effective approach to enhance postoperative recovery after thyroidectomy.

**Clinical Trial Registration:**

https://www.chictr.org.cn, identifier ChiCTR2400079949.

**First Trial Tegistration:**

01/17/2024.

## 1 Introduction

Postoperative sleep disturbances are common and are linked to impaired recovery and decreased patient satisfaction. Patients undergoing thyroidectomy are particularly vulnerable, often reporting significantly disrupted sleep during the first few nights after surgery (Maraş Baydoğan et al., 2025), even when analgesia is deemed adequate. Opioid-based patient-controlled intravenous analgesia (PCIA) remains a widely used strategy for postoperative pain management. However, opioids are linked to changes in sleep architecture and circadian rhythm regulation (Schiem et al., 2021; Dimsdale et al., 2007), and are further limited by side effects such as nausea, vomiting, and respiratory depression (Zhang et al., 2023). These drawbacks have prompted the development of opioid-sparing and opioid-free analgesic regimens that aim to provide effective pain control while supporting physiological recovery, including the preservation of normal sleep and circadian rhythms.

Melatonin, the primary hormone secreted by the pineal gland, plays a key role in regulating the sleep-wake cycle and circadian homeostasis (Xie et al., 2017; Comai and Gobbi, 2024; Drăgoi et al., 2026). Surgical stress and anesthetic exposure suppress nocturnal melatonin secretion (Guo et al., 2023; Naguib et al., 2007; Cronin et al., 2000), which may contribute to postoperative sleep disruption and has been linked to increased pain sensitivity and delayed cognitive recovery. Opioids may further exacerbate these disturbances, possibly through additional suppression of pineal melatonin release, thereby perpetuating a cycle of impaired melatonin signaling and disrupted sleep (Bumgarner et al., 2023; Mehranfard et al., 2025). Based on this, an opioid-free analgesic strategy may better preserve endogenous melatonin secretion, thereby supporting more restorative postoperative sleep.

Dexmedetomidine, a highly selective α_2_-adrenoceptor agonist, provides anxiolysis and analgesia with a sedative profile resembling physiological non-rapid eye movement sleep, and is associated with minimal respiratory depression (Ramaswamy et al., 2021; Kong et al., 2023). When combined with flurbiprofen axetil, a potent injectable nonsteroidal anti-inflammatory drug (NSAID), it forms a solid foundation for opioid-free PCIA by offering multimodal analgesia while reducing or eliminating opioid exposure (Rai et al., 2017). Although this combination has demonstrated promising analgesic efficacy and a favorable side effect profile in perioperative settings, its specific impact on perioperative melatonin dynamics and patient-reported sleep quality in thyroidectomy patients remains unclear.

Therefore, we conducted a randomized controlled trial to test the hypothesis that, compared with a conventional sufentanil-based PCIA regimen, an opioid-free PCIA regimen based on dexmedetomidine and flurbiprofen axetil would attenuate postoperative suppression of melatonin secretion (as reflected by urinary 6-sulfatoxymelatonin [aMT6s] excretion) (St Hilaire and Lockley, 2022; Lockley, 2020), improve subjective sleep quality, and reduce the incidence of opioid-related side effects in thyroidectomy patients.

## 2 Methods

### 2.1 Study design and ethics approval

This prospective, double-blind, randomized controlled trial was conducted in accordance with the ethical principles of the Declaration of Helsinki. The study protocol was approved by the Clinical Research Ethics Committee of the First Affiliated Hospital of Gannan Medical University (approval no. LLSC2023512) and was registered with the China Clinical Trial Registry (Registration No. ChiCTR2400079949) prior to patient enrollment. Written informed consent was obtained from all participants or their legal guardians.

### 2.2 Sample size calculation

The sample size was calculated *a priori* using PASS 2021 software. Based on previously reported Richards-Campbell Sleep Questionnaire (RCSQ) scores in patients undergoing comparable moderately invasive surgical procedures under opioid-based analgesia, including a study on elderly populations (Deng et al., 2023), the mean RCSQ score in the control group was assumed to be 70 with a standard deviation of 25. A between-group difference of 13 points in the RCSQ score was considered clinically meaningful, in accordance with prior clinical studies. With a two-sided α level of 0.05 and a statistical power of 80%, the estimated sample size required was 38 patients per group. Allowing for an anticipated dropout rate of 15%, a total sample size of 88 patients was planned. To ensure adequate statistical power, 96 patients were ultimately enrolled (48 per group).

Although one of the reference studies included elderly patients, it was used as a conservative reference because it reported RCSQ-based postoperative sleep outcomes under opioid-based analgesia. To further assess the robustness of the sample size assumption, we compared the assumed SD with the observed RCSQ variability in the present cohort. The observed SDs of RCSQ scores were 8.2–8.4 at baseline and 7.1–7.2 at the primary endpoint, which were lower than the assumed SD of 25. These findings suggest that the final sample size was adequate to detect the prespecified clinically meaningful difference of 13 points.

### 2.3 Patient selection

A total of 96 patients scheduled for elective thyroidectomy were screened and enrolled in this randomized controlled trial.

#### 2.3.1 Inclusion criteria

Patients were eligible for inclusion if they met all of the following criteria:

1. Age between 18 and 65 years;
2. Body mass index (BMI) between 18 and 30 kg/m^2^;
3. American Society of Anesthesiologists (ASA) physical status I or II.

#### 2.3.2 Exclusion criteria

Patients were excluded if they met any of the following criteria:

1. Severe cardiac, hepatic, or renal dysfunction;
2. Pre-existing sinus bradycardia, cardiac conduction abnormalities, or clinically significant arrhythmias;
3. Diagnosed psychiatric or neurological disorders, clinically relevant sleep disorders, or communication barriers that could interfere with sleep assessment or data collection;
4. Chronic use of opioid analgesics, sedative–hypnotic medications, antidepressants, or α_2_-adrenergic receptor agonists prior to surgery;
5. Known hypersensitivity or contraindications to NSAIDs, including flurbiprofen axetil, or a history of gastrointestinal ulceration or bleeding;
6. Intraoperative events requiring deviation from the standardized anesthesia or analgesia protocol, postoperative admission to the intensive care unit, or surgical site infection;
7. Patient withdrawal of consent, or loss or contamination of biological specimens resulting in incomplete or unreliable data.

### 2.4 Randomization, blinding, and group allocation

#### 2.4.1 Randomization

Eligible patients were randomly assigned in a 1:1 ratio to either the opioid-free group (OF group) or the opioid group (O group) using a computer-generated randomization sequence.

The randomization list was prepared by an independent statistician who was not involved in patient enrollment, perioperative management, or outcome assessment.

Group assignments were placed in sequentially numbered, opaque, sealed envelopes to ensure allocation concealment.

#### 2.4.2 Group allocation and interventions

Patients were allocated to one of the following patient-controlled intravenous analgesia (PCIA) regimens:

OF group: flurbiprofen axetil, dexmedetomidine, and ondansetron.

O group: sufentanil and ondansetron.

All other aspects of anesthesia management and perioperative care were standardized between the two groups.

Details regarding drug concentrations, PCIA pump preparation, and administration protocols are provided in Section 2.7.

Baseline demographic and clinical characteristics were comparable between groups (Table 1).

**TABLE 1:** Baseline demographic, clinical, and preoperative characteristics of patients by study group.

Characteristic	Opioid-free group (n = 47)	Opioid group (n = 48)	P value
Age (years)	41.0 ± 7.2	39.7 ± 6.3	0.360
Sex, n (%)			
Male	14 (29.8%)	13 (27.1%)	0.770
Female	33 (70.2%)	35 (72.9%)	
BMI (kg/m^2^)	22.84 ± 2.15	23.03 ± 2.30	0.680
ASA physical status, n (%)			
I	15 (31.9%)	16 (33.3%)	0.883
II	32 (68.1%)	32 (66.7%)	
Extent of thyroidectomy, n (%)			
Unilateral	35 (74.5%)	37 (77.1%)	0.814
Bilateral	12 (25.5%)	11 (22.9%)	
Room type, n (%)			
Single-bed room	2 (4.3%)	3 (6.3%)	0.890
Double-bed room	24 (51.1%)	23 (47.9%)	
Triple-bed room	21 (44.6%)	22 (45.8%)	
Operation time (min)	85.1 ± 16.4	82.7 ± 15.8	0.465
Education (years)	12.1 ± 3.5	12.5 ± 3.6	0.584
RCSQ score	63.0 ± 8.2	65.1 ± 8.4	0.549
SAS index score	50.4 ± 4.7	49.9 ± 4.6	0.953

Data are presented as mean ± standard deviation or number (percentage), as appropriate. BMI, body mass index; ASA, american society of anesthesiologists; RCSQ, Richards-Campbell Sleep Questionnaire; SAS, Self-Rating Anxiety Scale. Between-group comparisons were performed using independent-samples t tests or Mann–Whitney U tests for continuous variables, and χ^2^ tests or Fisher’s exact tests for categorical variables, as appropriate. A two-sided *P* < 0.05 was considered statistically significant.

#### 2.4.3 Blinding procedure

This study was conducted using a double-blind design.

The randomization envelope was opened solely by the anesthesiologist responsible for intraoperative management and preparation of the PCIA pump.

This anesthesiologist had no role in postoperative follow-up or outcome assessment.

To maintain blinding despite the milky-white appearance of flurbiprofen axetil, all prepared PCIA pumps were immediately placed into identical, opaque, sealed outer bags, making them visually indistinguishable.

As a result, patients, surgeons, ward nurses, and investigators responsible for postoperative assessments, data collection (RCSQ, VAS, SAS scores, adverse events), and outcome evaluation remained blinded to group allocation throughout the study. Although the infusion tubing was not specifically covered, it was routinely positioned beneath the patient’s clothing or bedding and was not visible to patients, ward nurses, or outcome assessors during postoperative follow-up, thereby maintaining the integrity of the blinding procedure.

### 2.5 Preoperative procedures and data collection

Prior to surgery, eligible patients were approached, screened, and enrolled in the study after providing written informed consent.

Baseline demographic and clinical information, including age, sex, height, weight, American Society of Anesthesiologists physical status, education level, medical history, and current medications, were recorded.

Baseline assessments were conducted on the morning of surgery.

Patients received standardized instructions on completing the RCSQ and completed the questionnaire independently under supervision.

To minimize circadian variability, patients were instructed on the collection of the first morning midstream urine sample.

Approximately 2 mL of urine from the first morning void (around 6:00 a.m.) was collected in sterile tubes and immediately stored at −80 °C for subsequent analysis of urinary 6-sulfatoxymelatonin.

### 2.6 Anesthesia protocol

All patients fasted for at least 8 h for solids and 2 h for clear fluids before surgery. Upon arrival in the operating room, standard monitoring was applied, including electrocardiography, non-invasive blood pressure, heart rate, respiratory rate, pulse oximetry, and end-tidal carbon dioxide monitoring. Bispectral index (BIS) monitoring was used to guide the depth of anesthesia.

General anesthesia was induced intravenously with midazolam (0.03 mg/kg), sufentanil (0.4–0.6 μg/kg), propofol (1.5–2.0 mg/kg), and rocuronium bromide (0.8 mg/kg) following preoxygenation. Tracheal intubation was performed using a video laryngoscope. Mechanical ventilation was initiated in volume-controlled mode with a tidal volume of 8–10 mL/kg, a respiratory rate of 10–15 breaths/min, and an inspiratory-to-expiratory ratio of 1:2, maintaining peak airway pressure below 30 cmH_2_O.

Anesthesia was maintained using a balanced technique with propofol (4–6 mg/kg/h), remifentanil (0.1–0.2 μg/kg/min), and sevoflurane (1–2 vol%), with dosages titrated to maintain BIS values between 40 and 60. Neuromuscular blockade was maintained with intermittent doses of rocuronium bromide. A supplemental dose of sufentanil (0.1 μg/kg) was administered intravenously before skin closure.

Approximately 5 min before the end of surgery, the patient-controlled intravenous analgesia (PCIA) pump was connected and initiated, after which all anesthetic infusions were discontinued according to standard practice.

A standardized protocol was used for the management of intraoperative adverse events to ensure hemodynamic stability:

Bradycardia (heart rate <50 beats/min) was treated with intravenous atropine (0.5 mg), with isoprenaline administered if unresponsive.

Tachycardia (heart rate >100 beats/min) was managed with esmolol after excluding insufficient anesthesia or analgesia.

Hypotension (mean arterial pressure decrease >20% from baseline) was treated with intravenous ephedrine (6 mg).

Hypertension (mean arterial pressure increase >20% from baseline) was treated with nitroglycerin after excluding reversible causes.

At the end of surgery, patients were transferred to the post-anesthesia care unit for routine monitoring. Tracheal extubation was performed after recovery of consciousness, adequate spontaneous ventilation (tidal volume >6 mL/kg), and sufficient neuromuscular function. Patients were subsequently returned to the ward with supplemental oxygen and standard postoperative monitoring.

### 2.7 Study intervention: PCIA formulation and management

#### 2.7.1 Preparation and formulation

The patient-controlled intravenous analgesia (PCIA) solutions were prepared under aseptic conditions by an anesthesiologist who was not involved in postoperative assessments, strictly according to the randomized group allocation. In the OF group, calculated doses of flurbiprofen axetil (4 mg/kg), dexmedetomidine (2 μg/kg), and ondansetron (16 mg) were combined. In the O group, the calculated dose of sufentanil (2 μg/kg) and ondansetron (16 mg) were combined. Each drug combination was diluted with 0.9% normal saline to a total volume of 100 mL and loaded into identical PCIA pump reservoirs.

#### 2.7.2 Administration parameters and protocol

All PCIA pumps were programmed with identical settings, including a continuous background infusion rate of 2 mL/h, a patient-controlled bolus dose of 2 mL, a lockout interval of 15 min, and a maximum allowable dose of 10 mL per hour. For dexmedetomidine, this corresponded to a background infusion dose of approximately 0.04 μg/kg/h in the OF group. The PCIA pump was connected and initiated approximately 5 min before the end of surgery. Postoperative pain control followed a predefined protocol: analgesia was considered adequate when the resting visual analog scale (VAS) score was ≤3. Patients were instructed to self-administer a bolus dose when the VAS score exceeded 3. The number of patient-initiated bolus attempts was recorded during each 24-h period as an indicator of supplemental analgesic demand.

#### 2.7.3 Management of analgesia-related adverse events

Standardized protocols were applied for the management of analgesia-related adverse events. Postoperative nausea and vomiting were treated with intravenous ondansetron (8 mg) as first-line rescue therapy. Patients were allowed to resume PCIA use after symptom resolution. Patients were routinely monitored for excessive sedation, respiratory depression, hypotension, and bradycardia. If clinically significant adverse events occurred, PCIA administration was temporarily suspended and managed according to institutional protocols.

### 2.8 Outcome measures and assessment time points

Outcome measures were assessed at predefined time points to evaluate the efficacy and safety of the study interventions.

#### 2.8.1 Primary outcome

Subjective sleep quality was assessed using the RCSQ. The primary endpoint was the RCSQ score on the first postoperative morning (T1), reflecting sleep quality during the first postoperative night.

RCSQ scores on the second postoperative morning (T2) were also collected to evaluate the persistence of postoperative sleep effects.

Baseline RCSQ values (T0) were obtained on the morning of surgery, referring to sleep quality on the night before surgery.

#### 2.8.2 Secondary outcomes

Secondary outcomes included the following measures:

1. Endogenous melatonin secretion: Nocturnal melatonin secretion was assessed by measuring urinary 6-sulfatoxymelatonin (6-SMT) in first-morning void urine samples collected at baseline (T0) and on postoperative days 1 (T1) and 2 (T2). Urinary 6-SMT concentrations were quantified using a commercially available enzyme-linked immunosorbent assay (ELISA) kit (ALPCO, Salem, NH, United States of America) according to the manufacturer’s instructions. Frozen urine samples were thawed at room temperature and centrifuged prior to analysis. All samples were measured in duplicate, and the mean value was used for statistical analysis. To account for variations in urine concentration, urinary 6-SMT levels were normalized to urinary creatinine concentrations and expressed as ng/mg creatinine.
2. Sleep parameters: Detailed sleep parameters were assessed during the first two postoperative nights using both patient-reported sleep diaries and a wrist-worn wearable device (Actiwatch Spectrum Plus, Philips Respironics, United States of America). Sleep parameters included sleep latency (defined as the time from lights-off to sleep onset), number of nocturnal awakenings (defined as awakenings lasting >5 min after sleep onset), and total sleep time (defined as the cumulative duration of sleep between sleep onset and final morning awakening, excluding periods of wakefulness). Baseline sleep parameters (T0) were recorded for comparison.
3. Anxiety level: Anxiety was evaluated using the Zung Self-Rating Anxiety Scale (SAS), consisting of 20 items scored on a four-point Likert scale, with items 5, 9, 13, 17, and 19 reverse-scored. The raw score (range 20–80) was converted to the SAS Index score by multiplying by 1.25 (range 25–100). SAS Index scores were assessed at baseline (T0) and on postoperative days 1 (T1) and 2 (T2).
4. Postoperative pain intensity: Postoperative pain at rest was assessed using the visual analog scale (VAS) at 1, 6, 24, and 48 h after surgery.
5. Sedation level: Sedation was evaluated using the Ramsay Sedation Scale at 1, 6, 24, and 48 h postoperatively.
6. Adverse events:The incidence of postoperative nausea and vomiting, pruritus, dizziness, respiratory depression (respiratory rate <8 breaths/min or SpO_2_<90%), bradycardia (heart rate <50 beats/min), surgical site bleeding, and acute kidney injury was actively monitored and recorded during the first 48 postoperative hours.

#### 2.8.3 Blinding of outcome assessment

All postoperative outcome assessments (T0, T1, T2, and hourly time points) were performed by trained research staff who remained blinded to group allocation, as described in Section 2.4.

### 2.9 Statistical analysis

Statistical analyses were performed using SPSS software (version 26.0; IBM Corp., Armonk, NY, United States). Data presentation and figure generation were conducted with GraphPad Prism (version10.0; GraphPad Software, San Diego, CA, United States). Continuous variables are presented as mean ± standard deviation (SD) or median (interquartile range) based on their distribution, as assessed by the Shapiro—Wilk test. Categorical variables are presented as numbers (percentages).

The primary analysis followed the intention-to-treat principle, including all randomized patients in the groups to which they were originally assigned. Between-group comparisons of the primary outcome (RCSQ score at T1) and other continuous secondary outcomes were performed using independent samples *t*-tests or Mann-Whitney U tests, as appropriate. For outcomes measured at multiple time points (e.g., VAS, Ramsay scores), a two-way repeated-measures analysis of variance (ANOVA) was employed, with time and group as factors, followed by post-hoc tests with Bonferroni correction for multiple comparisons. Categorical data, including the incidence of adverse events, were compared using the Chi-square test or Fisher’s exact test. A two-sided P value < 0.05 was considered statistically significant.

## 3 Results

### 3.1 Participant flow and baseline characteristics

**FIGURE 1:**
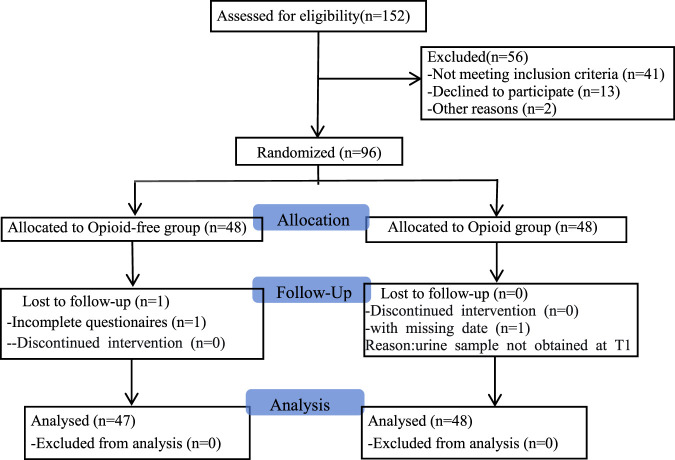
CONSORT flow diagram.

From Janaury 26, 2024 to December 26, 2024, a total of 152 patients were screened for eligibility. Of these, 96 patients were enrolled and randomly assigned to either the opioid-free (OF) group (n = 48) or the opioid-based (O) group (n = 48). The flow of participants through the study is shown in Figure 1.

During the follow-up period, one patient in the OF group was excluded from questionnaire-based analyses because of incomplete RCSQ data, and one patient in the O group was excluded from urinary melatonin analysis at T2 owing to a missing urine specimen. All 96 randomized patients were included in the primary intention-to-treat analysis.

Baseline demographic and clinical characteristics were comparable between the two groups, as summarized in Table 1.

### 3.2 Primary outcome: postoperative sleep quality

The primary outcome, postoperative sleep quality assessed by the RCSQ on the first postoperative morning (T1), was significantly higher in the OF group than in the O group (68.1 ± 7.1 vs. 59.8 ± 7.2; mean difference, 8.4; 95% CI, 4.8–11.9; *P* < 0.001). This between-group difference in sleep quality remained significant on the second postoperative morning (T2) (75.8 ± 5.4 vs. 61.1 ± 6.0; *P* < 0.001).

**FIGURE 2:**
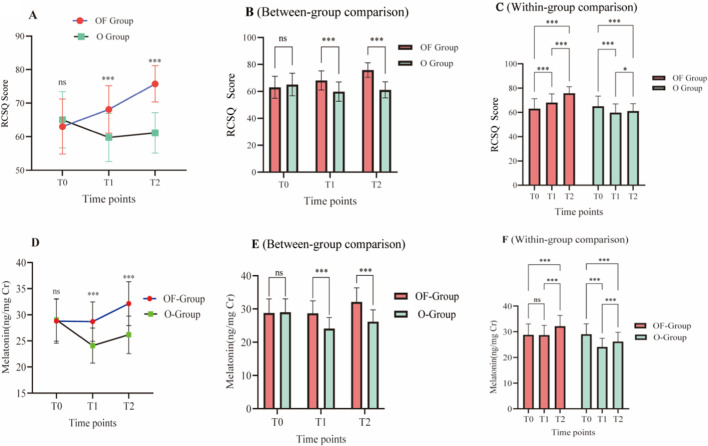
Perioperative changes in sleep quality assessed by the Richards-Campbell Sleep Questionnaire (RCSQ) and urinary melatonin secretion in patients undergoing thyroidectomy. **(A)** Longitudinal changes in RCSQ scores at the preoperative night (T0), postoperative day 1 night (T1), and postoperative day 2 night (T2) in the OF group and the O group. **(B)** Between-group comparisons of RCSQ scores at each time point. **(C)** Within-group comparisons of RCSQ scores across perioperative time points. **(D)** Longitudinal changes in morning urinary 6-sulfatoxymelatonin levels corrected for creatinine (6-SMT/Cr). **(E)** Between-group comparisons of urinary 6-SMT/Cr at each time point. **(F)** Within-group comparisons of urinary 6-SMT/Cr across perioperative time points. Data are presented as mean ± standard deviation. Statistical analyses were performed using repeated-measures analysis of variance. Post hoc pairwise comparisons were conducted with Bonferroni correction for multiple comparisons. Between-group comparisons were performed at each time point, and within-group comparisons were performed across perioperative time points. T0, preoperative night; T1, postoperative day 1 night; T2, postoperative day 2 night.^*^ *P* < 0.05, ^**^ *P* < 0.01, ^***^ *P* < 0.001; ns, not significant.

Changes in RCSQ scores over time, together with urinary 6-SMT levels, are presented in Figure 2 and Table 2.

**TABLE 2:** Perioperative changes in sleep quality, melatonin secretion, and anxiety levels.

Outcome	Time point	Opioid-free group (n = 47)	Opioid group (n = 48)	*P* value (between group)
RCSQ total score	T0	63.0 ± 8.2	65.1 ± 8.4	0.549
	T1	68.1 ± 7.1	59.8 ± 7.2	<0.001
	T2	75.8 ± 5.4	61.1 ± 6.0	<0.001
Urinary 6-sulfatoxymelatonin (6-SMT/Cr, ng/mg)	T0	28.79 ± 4.22	29.01 ± 4.05	0.992
	T1	28.69 ± 3.77	24.10 ± 3.35	<0.001
	T2	32.13 ± 4.20	26.17 ± 3.59 (n = 47)	<0.001
SAS index score	T0	50.4 ± 4.7	49.9 ± 4.6	0.953
	T1	48.2 ± 4.3	52.0 ± 4.2	<0.001
	T2	42.9 ± 3.6	49.3 ± 4.2	<0.001

Data are presented as mean ± standard deviation. T0, preoperative night; T1, postoperative day 1 night; T2, postoperative day 2 night. RCSQ, Richards-Campbell Sleep Questionnaire; 6-SMT/Cr, urinary 6-sulfatoxymelatonin corrected for creatinine; SAS, Self-Rating Anxiety Scale. Between-group comparisons at each time point were performed using independent-samples t tests (or Mann–Whitney U tests, as appropriate). One patient in the opioid group was excluded from urinary melatonin measurements at T2 due to a missing urine sample. A two-sided *P* < 0.05 was considered statistically significant.

### 3.3 Secondary outcomes: melatonin secretion

Consistent with the observed improvements in postoperative sleep quality, nocturnal melatonin secretion was better preserved in the OF group. Urinary 6-SMT levels, normalized to creatinine, did not differ between groups at baseline (T0).

On the first postoperative morning (T1), urinary 6-SMT was significantly higher in the OF group than in the O group (28.69 ± 3.77 vs. 24.10 ± 3.35 ng/mg Cr, *P* < 0.001). This between-group difference remained significant on the second postoperative morning (T2) (32.13 ± 4.20 vs. 26.17 ± 3.59 ng/mg, *P* < 0.001).

### 3.4 Secondary outcomes: sleep parameters

**FIGURE 3:**
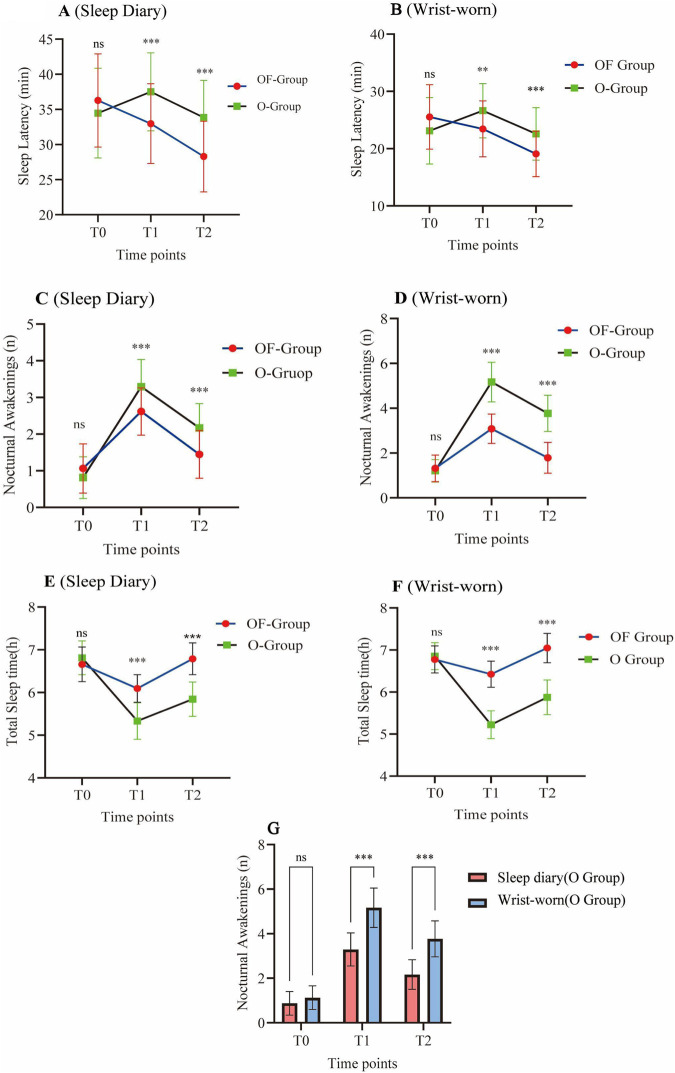
Changes in detailed sleep parameters assessed by sleep diaries and a wrist-worn device. Figure 3 shows perioperative changes in detailed sleep parameters assessed using patient-reported sleep diaries and a wrist-worn wearable device in the opioid-free (OF) and opioid (O) groups. Sleep latency is shown in **(A)** (sleep diary) and **(B)** (wrist-worn device), nocturnal awakenings in **(C)** (sleep diary) and **(D)** (wrist-worn device), and total sleep time in **(E)** (sleep diary) and **(F)** (wrist-worn device), at baseline (T0), postoperative day 1 (T1), and postoperative day 2 (T2). Across postoperative time points, both assessment methods demonstrated consistent between-group differences, with the OF group exhibiting shorter sleep latency, fewer nocturnal awakenings, and longer total sleep time at T1 and T2, while no significant differences were observed at T0. **(G)** compares nocturnal awakenings recorded by sleep diaries and the wrist-worn device within the opioid group, showing a higher frequency detected by the wearable device at T1 and T2 but not at T0. Data are presented as mean ± standard deviation. Statistical analyses for **(A‐F)** were performed using repeated-measures analysis of variance, with group and time as factors. Post hoc pairwise comparisons were conducted with Bonferroni correction for multiple comparisons. For **(G)**, comparisons between sleep diary- and wrist-worn device-detected awakenings within the opioid-based group at each time point were performed using paired-samples t tests, with Bonferroni correction for multiple comparisons. T0, preoperative night; T1, postoperative day 1 night; T2, postoperative day 2 night.^*^ *P* < 0.05, ^**^ *P* < 0.01, ^***^ *P* < 0.001; ns, not significant.

Detailed sleep parameters assessed using patient-reported sleep diaries and a wrist-worn wearable device are shown in Figure 3. Both assessment methods demonstrated consistent between-group differences in sleep latency, nocturnal awakenings, and total sleep time across postoperative time points. Compared with the O group, patients in the OF group exhibited shorter sleep latency and longer total sleep time on postoperative days 1 (T1) and 2 (T2) as assessed by both sleep diaries and the wrist-worn wearable device (all *P* < 0.05). Baseline sleep parameters (T0) did not differ significantly between groups.

For nocturnal awakenings, both diary-based and wearable-derived data showed a higher frequency of awakenings in the O group than in the OF group at T1 and T2 (all *P* < 0.05). Notably, the number of nocturnal awakenings recorded by the wrist-worn wearable device was consistently higher than that reported in sleep diaries within the O group (*P* < 0.05), whereas no significant difference between the two assessment methods was observed at baseline (T0). For further details, please refer to Figure 3.

### 3.5 Secondary outcomes: anxiety levels

Postoperative anxiety, assessed using the SAS Index score, differed significantly between groups after surgery. Baseline SAS Index scores (T0) were comparable between the OF and O groups.

On postoperative day 1 (T1), the SAS Index score was significantly lower in the OF group than in the O group (48.2 ± 4.3 vs. 52.0 ± 4.2, *P* < 0.001). This between-group difference persisted on postoperative day 2 (T2), with further reductions observed in the opioid-free group (42.9 ± 3.6 vs. 49.3 ± 4.2, *P* < 0.001). Detailed data are presented in Table 2.

### 3.6 Analgesic efficacy and sedation

Postoperative analgesic efficacy was comparable between the two groups. Resting pain intensity, assessed by VAS scores, did not differ significantly between the OF and O groups at any postoperative time point. Sedation levels, evaluated using the Ramsay Sedation Scale, were also similar between groups throughout the 48-h postoperative period. In addition, the total number of patient-initiated PCIA bolus attempts over 48 h did not differ significantly between groups. Detailed data are presented in Table 3.

**TABLE 3:** Postoperative pain intensity and sedation levels.

Outcome	Time point	Opioid-free group (n = 47)	Opioid group (n = 48)	*P* value
VAS score at rest	1 h	2 (2.3)	3 (2.3)	0.115
	6 h	2 (2.3)	2 (2.3)	0.213
	24 h	2 (1.2)	2 (2.3)	0.503
	48 h	1 (1.2)	2 (2.2)	0.230
Sedation (ramsay score)	1 h	2 (1.2)	2 (1.2)	0.089
	6 h	1 (1.1)	1 (1.1)	0.314
	24 h	1 (1.1)	1 (1.1)	0.988
	48 h	1 (1.1)	1 (1.1)	0.325
Total PCA bolus attempts (48 h)		2 (1.2)	2 (1.2)	0.611

Data are presented as median (interquartile range). VAS, visual analog scale; PCA, patient-controlled analgesia. Sedation was assessed using the Ramsay Sedation Scale. Between-group comparisons at each time point were performed using the Mann–Whitney U test. A two-sided *P* < 0.05 was considered statistically significant.

### 3.7 Safety outcomes: adverse events

The incidence of postoperative adverse events within 48 h is summarized in Table 4. Overall, both analgesic regimens were well tolerated, and no serious adverse events were observed in either group.

**TABLE 4:** Incidence of postoperative adverse events within 48 h.

Adverse event	Opioid-free group (n = 47)	Opioid group (n = 48)	P value
Opioid-related events			
Nausea	3 (6.4%)	10 (22.9%)	0.023
Vomiting	2 (4.3%)	8 (16.7%)	0.049
Pruritus	1 (2.1%)	7 (14.6%)	0.029
Respiratory depression	0 (0%)	0 (0%)	-
Opioid-free regimen-related events			
Dexmedetomidine-related events			​
Bradycardia	6 (12.8%)	2 (4.2%)	0.228
Hypotension	4 (8.5%)	1 (2.1%)	0.168
NSAID-associated (flurbiprofen axetil)			
Surgical site bleeding	0 (0%)	0 (0%)	**-**
Acute kidney injury	0 (0%)	0 (0%)	**-**
Other/ Non-specific events			
Dizziness	3 (6.4%)	7 (14.6%)	0.205

Data are presented as number (percentage). Between-group comparisons were performed using χ^2^ tests or Fisher’s exact tests, as appropriate. NSAID, nonsteroidal anti-inflammatory drug. A two-sided *P* < 0.05 was considered statistically significant.

Opioid-related adverse events, including nausea, vomiting, and pruritus, occurred significantly less frequently in the OF group than in the O group (all *P* < 0.05). No episodes of respiratory depression were recorded in either group.

Dexmedetomidine-related adverse events, including bradycardia and hypotension, were infrequent and did not differ significantly between groups. No NSAID-associated complications, such as surgical site bleeding or acute kidney injury, were observed in either group. The incidence of other nonspecific adverse events, including dizziness, was comparable between groups. For detailed information, please refer to Table 4.

## 4 Discussion

The primary finding of this study is that, in patients undergoing thyroidectomy, a non-opioid multimodal analgesic regimen based on dexmedetomidine and flurbiprofen axetil provided postoperative analgesia comparable to opioid-based (sufentanil) PCIA, while significantly improving postoperative sleep quality, as reflected by higher RCSQ scores. Importantly, this sleep benefit was accompanied by better preservation of endogenous melatonin secretion: postoperative morning urinary 6-SMT levels were not suppressed in the non-opioid group and remained consistently higher than those in the opioid group. These findings support the hypothesis that non-opioid multimodal analgesia may be associated with better preservation of circadian melatonin secretion and improved postoperative sleep quality.

The preservation of postoperative 6-SMT levels in the non-opioid group is a key finding. Traditionally, surgical stress and the hospital environment are known to suppress melatonin secretion (Cronin et al., 2000; Gögenur, 2010). However, our data suggest that an appropriate analgesic-sedative strategy can counteract this tendency. Several mechanisms may contribute to this effect. First, avoiding direct suppression: opioids such as sufentanil act on μ-opioid receptors in the pineal gland, inhibiting melatonin synthesis (Esposti et al., 1988). By avoiding opioids, the non-opioid regimen circumvents this pathway. Second, the circadian-protective effect of dexmedetomidine: while α2-adrenergic activation by dexmedetomidine might theoretically influence melatonin pathways (Zhang et al., 2024), its sympatholytic and anxiolytic properties attenuate surgical stress responses, further promoting sleep (Sui et al., 2022; Zeng et al., 2026). Additionally, dexmedetomidine induces a sedative state resembling physiological sleep (Ji et al., 2024). In addition to being associated with better preservation of circadian melatonin secretion, the non-opioid regimen’s anxiolytic effects also play a crucial role in enhancing sleep quality, further supporting its multifaceted benefits. Consistent with this, both sleep diary and wearable device data in our study demonstrated shorter sleep latency, fewer nocturnal awakenings, and longer total sleep time in the non-opioid group. Sleep continuity is a key determinant of melatonin rhythmicity, and melatonin’s physiological roles extend beyond circadian regulation to include anti-inflammatory and antioxidant actions (Jia et al., 2025). Furthermore, the anti-inflammatory synergy of flurbiprofen axetil may contribute to this effect (Huang et al., 2025), as COX inhibition stabilizes the internal milieu, promoting circadian stability (Cheng et al., 2024; Xu et al., 2021).

In addition, the non-opioid regimen was associated with significantly lower postoperative anxiety scores, as measured by the SAS. Postoperative anxiety correlates with sleep disturbances, and dexmedetomidine’s anxiolytic effects likely contribute to the observed improvement in sleep quality. By reducing both preoperative and postoperative anxiety (Xing et al., 2024; Qiu et al., 2024), dexmedetomidine enhances the overall sleep experience, suggesting that some sleep benefits of the non-opioid regimen may be due to its anxiolytic properties. This further underscores the value of non-opioid multimodal analgesia in improving patient outcomes beyond pain control.

An additional notable observation was the discrepancy between nocturnal awakenings detected by wrist-worn devices and those reported in sleep diaries in the opioid group, highlighting a perception-reality dissociation. This paradox may reflect a dissociation between objectively measured and subjectively perceived sleep disruptions in patients receiving opioid-based analgesia. One possible explanation is that the sedative effects of opioids reduce patients’ awareness of nocturnal awakenings; however, the present study was not designed to formally evaluate this mechanism (Tripathi et al., 2020; Nelson et al., 2025). As a result, opioid users may experience objectively impaired sleep that is not fully perceived subjectively, suggesting the potential underestimation of opioid-related sleep disruptions when relying solely on subjective reports (Schneider et al., 2024).

Furthermore, the lower incidence of postoperative nausea and vomiting (PONV) in the non-opioid group may ly explain the improved sleep quality observed, as PONV itself disrupts sleep. The antiemetic properties of dexmedetomidine likely contributed to this reduction (Li et al., 2022; Zhao et al., 2023). These findings suggest that the sleep advantages of the non-opioid regimen result from a combination of enhanced physiological sleep preservation and reduced opioid-related side effects.

Regarding hemodynamic safety, the background infusion of dexmedetomidine in the OF group corresponded to approximately 0.04 μg/kg/h. Although bradycardia and hypotension were not statistically different between groups, numerically more episodes occurred in the OF group, which may be related to the sympatholytic effect of dexmedetomidine. These events were infrequent, clinically manageable, and not associated with serious adverse outcomes. Nevertheless, careful hemodynamic monitoring remains necessary when dexmedetomidine-based opioid-free PCIA is used.

The study’s strength lies in its integrated approach, simultaneously evaluating objective circadian biomarkers (6-SMT), objective and subjective sleep parameters, and overall sleep quality (RCSQ) within a unified analytical framework. Our findings suggest a possible association among non-opioid multimodal analgesia, better preservation of circadian melatonin secretion, improved objective sleep continuity, and enhanced subjective sleep quality. Preserved melatonin rhythmicity may not only facilitate sleep initiation and maintenance but may also improve postoperative recovery through its anti-inflammatory and immunomodulatory effects.

This study has several limitations. First, the single-center design and the relatively homogeneous patient population (thyroidectomy patients) may limit the generalizability to more diverse surgical populations. Second, although we measured a stable melatonin metabolite, direct real-time monitoring of pineal activity or plasma melatonin was not performed. Third, the observational nature of the biomarker-sleep association prevents definitive causal conclusions. Therefore, causality or mediation between melatonin preservation and improved sleep quality cannot be established from the present data. Fourth, the observed discrepancy between device-detected and diary-reported awakenings should be interpreted cautiously, as formal interaction analyses were not performed and the study was not specifically designed to investigate mechanisms underlying sleep perception.

In summary, this study provides novel evidence linking non-opioid multimodal analgesia with preserved circadian melatonin rhythmicity and superior multidimensional sleep outcomes after thyroidectomy. Our findings emphasize the holistic approach to postoperative recovery, advocating for the integration of sleep and circadian rhythm management into Enhanced Recovery After Surgery (ERAS) protocols. Future research should validate these results in larger, multicenter trials and across different types of surgeries. Additionally, exploring whether early postoperative sleep parameters or 6-SMT levels can predict long-term recovery milestones (Abeysuriya et al., 2018), and employing advanced neurophysiological techniques (e.g., EEG) to assess dexmedetomidine’s effects on sleep microarchitecture, will be crucial next steps.

## Data Availability

The raw data supporting the conclusions of this article will be made available by the authors, without undue reservation.
